# Supplementary material for: The relationship between the gut microbiome and the risk of respiratory infections among newborns
Source: Commun Med (Lond). 2022 Jul 14;2:87. doi: 10.1038/s43856-022-00152-1 (PMC9283516; doi:10.1038/s43856-022-00152-1)
Supplement: Supplementary file 1 — Description of Additional Supplementary Files [file 43856_2022_152_MOESM1_ESM.pdf]

Description of Additional Supplementary Files

File Name: Supplementary Data 1

Description: Adjusted relative risk estimates, 95% confidence intervals, and number of outcomes from GEE analysis of 6-week-old infant stool 16S V4-V5 rRNA sequencing alpha diversity and infections and symptoms of infection over the first year of life

File Name: Supplementary Data 2

Description: Adjusted relative risk estimates, standard error, and p-value of the number of infections and symptoms over the first year of life in relation to 6-week metagenomics species relative abundance

File Name: Supplementary Data 3

Description: Adjusted relative risk estimates and 95% confidence intervals of metagenomics species associated with the number of infections and symptoms of infections in the first year of life
