# Supplementary material for: The relationship between the gut microbiome and the risk of respiratory infections among newborns
Source: Commun Med (Lond). 2022 Jul 14;2:87. doi: 10.1038/s43856-022-00152-1 (PMC9283516; doi:10.1038/s43856-022-00152-1)
Supplement: Supplementary file 5 — Supplementary Information [file 43856_2022_152_MOESM5_ESM.pdf]

The relationship between the gut microbiome and the risk of respiratory infections among newborns

Yuka Moroishi[1,2*], Jiang Gui[2], Anne G. Hoen[1,2], Hilary G. Morrison[3], Emily R. Baker[4], Kari Nadeau[5], Hongzhe Li[6], Zhigang Li[7], Juliette Madan[1,8], Margaret R. Karagas[1*]

[1]Department of Epidemiology, Geisel School of Medicine at Dartmouth, Hanover, New Hampshire, USA

[2]Department of Biomedical Data Science, Geisel School of Medicine at Dartmouth, Lebanon, New Hampshire, USA

[3]Josephine Bay Paul Center for Comparative Molecular Biology and Evolution, Marine Biological Laboratory, Woods Hole, Massachusetts, USA

[4]Department of Obstetrics and Gynecology, Dartmouth-Hitchcock Medical Center, Lebanon, New Hampshire, USA

[5]Sean N. Parker Center for Allergy and Asthma Research, Stanford University School of Medicine, Stanford, California, USA

[6]Department of Biostatistics, Epidemiology and Informatics, Perelman School of Medicine, University of Pennsylvania, Philadelphia, Pennsylvania, USA

[7]Department of Biostatistics, University of Florida, Gainesville, Florida, USA

[8]Department of Pediatrics, Children's Hospital at Dartmouth, Lebanon, New Hampshire, USA

*Corresponding Author

Supplementary Information

a) Heat map of 20 most common genera from 16S sequencing data (N = 465)

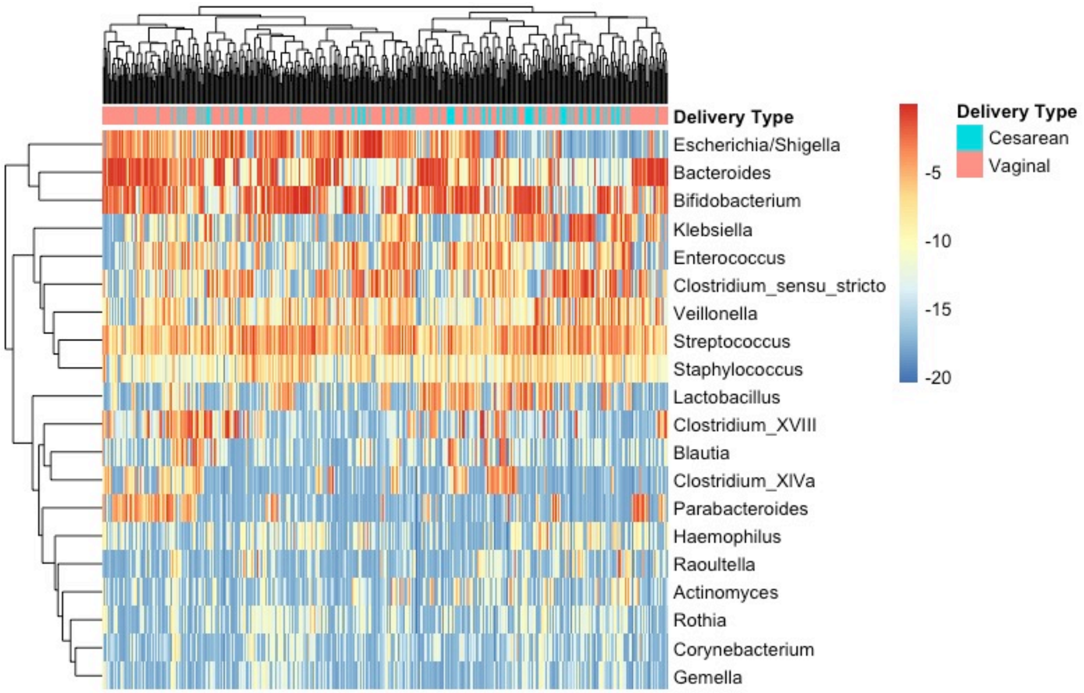

b) Heat map of 20 most common species from metagenomics sequencing data (N = 185)

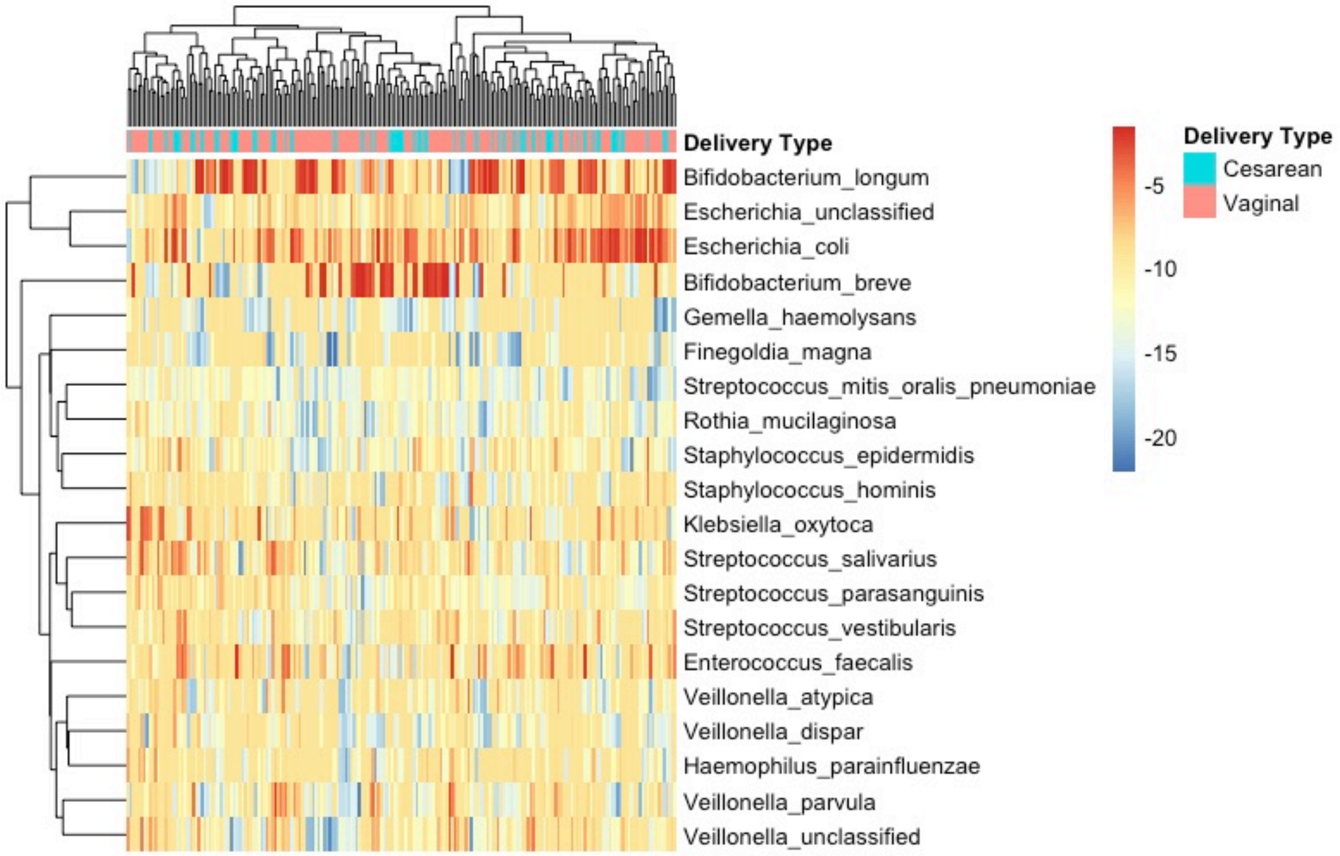

Supplementary Figure 1. Composition of 6-week Gut Microbiome of Infants in the NHBCS.

Supplementary Table 1. Number of Immune-related Outcomes over 12 Months at Each Time Period for 16S Analyses[a]

Outcome	Time Period	Overall N = 465	Vaginal Delivery N = 339	Cesarean Delivery N = 126
Any Infections or Symptoms	4 months	55	37	18
	8 months	189	133	56
	12 months	244	200	44
Upper Respiratory Infections	4 months	30	21	9
	8 months	109	79	30
	12 months	156	126	30
Lower Respiratory Infections	4 months	5	4	1
	8 months	26	18	8
	12 months	18	16	2
Wheezing	4 months	4	2	2
	8 months	19	14	5
	12 months	20	16	4
Diarrhea	4 months	7	5	2
	8 months	11	9	2
	12 months	18	13	5

[a] For any infections or symptoms, 18 entries were imputed. For upper RTI, 3 entries were imputed. For lower RTI, 3 entries were imputed.

Supplementary Table 2. Number of Immune-related Outcomes over 12 Months at Each Time Period for Metagenomics Analyses[a]

Outcome	Time Period	Overall N = 185	Vaginal Delivery N = 129	Cesarean Delivery N = 26
Any Infections or Symptoms	4 months	16	11	5
	8 months	72	42	30
	12 months	119	96	23
Upper Respiratory Infections	4 months	12	10	2
	8 months	48	31	17
	12 months	72	58	14
Lower Respiratory Infections	4 months	0	0	0
	8 months	6	3	3
	12 months	8	7	1
Wheezing	4 months	1	0	1
	8 months	6	3	3
	12 months	11	8	3
Diarrhea	4 months	3	2	1
	8 months	4	3	1
	12 months	7	5	2

[a] For any infections or symptoms, 7 entries were imputed.

Supplementary Table 3. Adjusted Relative Risk Estimates and 95% Confidence Intervals from GEE Analysis of Infant 6-Week Stool 16S V4-V5 rRNA Sequencing Alpha Diversity and Infections and Symptoms of Infection over the First Year of Life [a]

Outcome	Overall N = 465			Vaginal Delivery N = 339			Cesarean Delivery N = 126		
	No. of Outcomes[b]	RR	95% CI	No. of Outcomes[b]	RR	95% CI	No. of Outcomes[b]	RR	95% CI
Any Infection or Symptom[c]	488	1.39*	(1.1, 1.77)	370	1.62*	(1.23, 2.15)	118	0.95	(0.58, 1.55)
Upper RTI	295	1.40*	(1.12, 1.76)	226	1.85	(0.98, 3.51)	69	0.94	(0.27, 3.29)
Lower RTI	49	1.50	(0.87, 2.6)	38	1.89	(0.96, 3.72)	11	0.94	(0.28, 3.21)
Wheezing	43	1.30	(0.81, 2.07)	32	2.00*	(1.16, 3.45)	11	0.47	(0.20, 1.12)
Diarrhea[d]	36	1.44	(0.92, 2.25)	27	1.86*	(1.14, 3.03)	9	0.74	(0.32, 1.69)

Abbreviations: GEE, generalized estimating equation; N, sample size; No., number; RR, relative risk; CI, confidence interval; RTI, respiratory tract infection.

*indicates statistical significance at $\alpha = 0.05$.

[a] Overall GEE adjusted for maternal BMI, delivery type, sex, breast feeding at six weeks, perinatal antibiotic use, and gestational age. GEE stratified by delivery mode (vaginal and cesarean) adjusted for maternal BMI, sex, breast feeding at six weeks, perinatal antibiotic use, and gestational age. Relative risk estimates represent an increased risk of having an additional infection or symptom of infection or an increased risk of experiencing wheezing or diarrhea with each doubling of the inverse Simpson index. Upper RTI, lower RTI, and wheezing outcomes are those diagnosed by a physician for which a medication was prescribed. Diarrhea outcomes are those diagnosed by a physician for which no medication was prescribed.

[b] Total number of outcomes may be greater than N due to repeated measures.

[c] Any infection or symptom is the sum of upper respiratory tract infections (RTI), lower RTI, and acute respiratory symptoms.

[d] Sample sizes N = 464 for overall and N = 125 for cesarean delivery for diarrhea analyses due to missing data.
