# Supplementary material for: The relationship between the gut microbiome and the risk of respiratory infections among newborns
Source: Commun Med (Lond). 2022 Jul 14;2:87. doi: 10.1038/s43856-022-00152-1 (PMC9283516; doi:10.1038/s43856-022-00152-1)
Supplement: Supplementary file 7 — Reporting Summary [file 43856_2022_152_MOESM7_ESM.pdf]

nature research

Corresponding author(s): Yuka Moroishi

Last updated by author(s): 06/21/2022

Reporting Summary

Nature Research wishes to improve the reproducibility of the work that we publish. This form provides structure for consistency and transparency in reporting. For further information on Nature Research policies, see our Editorial Policies and the Editorial Policy Checklist.

Statistics

For all statistical analyses, confirm that the following items are present in the figure legend, table legend, main text, or Methods section.

n/a	Confirmed	
☐	☒	The exact sample size (n) for each experimental group/condition, given as a discrete number and unit of measurement
☐	☒	A statement on whether measurements were taken from distinct samples or whether the same sample was measured repeatedly
☐	☒	The statistical test(s) used AND whether they are one- or two-sided<br>*Only common tests should be described solely by name; describe more complex techniques in the Methods section.*
☐	☒	A description of all covariates tested
☐	☒	A description of any assumptions or corrections, such as tests of normality and adjustment for multiple comparisons
☐	☒	A full description of the statistical parameters including central tendency (e.g. means) or other basic estimates (e.g. regression coefficient) AND variation (e.g. standard deviation) or associated estimates of uncertainty (e.g. confidence intervals)
☐	☒	For null hypothesis testing, the test statistic (e.g. F, t, r) with confidence intervals, effect sizes, degrees of freedom and P value noted<br>*Give P values as exact values whenever suitable.*
☒	☐	For Bayesian analysis, information on the choice of priors and Markov chain Monte Carlo settings
☒	☐	For hierarchical and complex designs, identification of the appropriate level for tests and full reporting of outcomes
☐	☒	Estimates of effect sizes (e.g. Cohen's d, Pearson's r), indicating how they were calculated

Our web collection on statistics for biologists contains articles on many of the points above.

Software and code

Policy information about availability of computer code

Data collection	No software or code was used to collect data for this study.
Data analysis	All analyses were performed using R version 3.4.3 using the functions diversity, mice, and geeglm in the 'vegan', 'mice', and 'geepack' packages. DNA reads were merged and trimmed using KneadData for quality control before species-level taxonomic profiles were generated using Metaphlan. ASVs were inferred using DADA using the SILVA database.

For manuscripts utilizing custom algorithms or software that are central to the research but not yet described in published literature, software must be made available to editors and reviewers. We strongly encourage code deposition in a community repository (e.g. GitHub). See the Nature Research guidelines for submitting code & software for further information.

Data

Policy information about availability of data

All manuscripts must include a data availability statement. This statement should provide the following information, where applicable:

- Accession codes, unique identifiers, or web links for publicly available datasets
- A list of figures that have associated raw data
- A description of any restrictions on data availability

The microbiome data used in this study is publically available in the Sequence Read Archive at http://www.ncbi.nlm.nih.gov/sra under the accession number PRJNA296814. The full study data is not publicly available due to their sensitive and identifiable nature; it may be made available upon request to the corresponding author. The source data underlying Figures 1, 2 and 3 are in Supplementary Data 1, Supplementary Data 2, and Supplementary Data 3, respectively.

Field-specific reporting

Please select the one below that is the best fit for your research. If you are not sure, read the appropriate sections before making your selection.

☒ Life sciences ☐ Behavioural & social sciences ☐ Ecological, evolutionary & environmental sciences

For a reference copy of the document with all sections, see nature.com/documents/nr-reporting-summary-flat.pdf

Life sciences study design

All studies must disclose on these points even when the disclosure is negative.

Sample size	Sample size was chosen according to the number of participants in our prospective cohort that provided stool samples. The number is sufficient due to the statistical models we use to analyze high dimensional microbiome data.
Data exclusions	Participants were excluded from this study if that had not provided stool samples at six weeks of life and if they did not provide health outcome information during telephone surveys in the first year of life.
Replication	All findings are reproducible as determined by multiple runs of R code on the data.
Randomization	Allocation of subjects were not random due to our prospective cohort study design. However, all models were adjusted for confounding. These variables included maternal prepregnancy BMI (kg/m2), delivery mode (vaginal/cesarian), infant sex (male/female), breast feeding at six weeks (exclusively breastfed/mixed fed or exclusively formula fed), antibiotic use during pregnancy (yes/no), and gestational age (completed weeks)
Blinding	All data provided to authors for this analysis was deidentified.

Reporting for specific materials, systems and methods

We require information from authors about some types of materials, experimental systems and methods used in many studies. Here, indicate whether each material, system or method listed is relevant to your study. If you are not sure if a list item applies to your research, read the appropriate section before selecting a response.

Materials & experimental systems

n/a	Involved in the study	
☒	☐	Antibodies
☒	☐	Eukaryotic cell lines
☒	☐	Palaeontology and archaeology
☒	☐	Animals and other organisms
☐	☒	Human research participants
☒	☐	Clinical data
☒	☐	Dual use research of concern

Methods

n/a	Involved in the study	
☒	☐	ChIP-seq
☒	☐	Flow cytometry
☒	☐	MRI-based neuroimaging

Human research participants

Policy information about studies involving human research participants

Population characteristics	Participants included mother-infant dyads from the NHBCS from whom we obtained infant stool samples at approximately six weeks of age. Our study population had an approximately equal distribution of male (53.4%) and female infants (46.6%). Nearly half of the infants (56.2%) had been exclusively breastfed at approximately six weeks of age, and approximately one-fifth of mothers (18.5%) had reported antibiotic use during pregnancy. Cesarean section deliveries accounted for one-third of deliveries (30.3%).
Recruitment	Pregnant women aged 18 to 45 were recruited from prenatal clinics in New Hampshire, USA, starting in January 2009.
Ethics oversight	The Committee for the Protection of Human Subjects at Dartmouth College approved all protocols, and participants were provided with written informed consent upon enrollment.

Note that full information on the approval of the study protocol must also be provided in the manuscript.
